# Directed Differentiation of Human Embryonic Stem Cells into Prostate Organoids *In Vitro* and its Perturbation by Low-Dose Bisphenol A Exposure

**DOI:** 10.1371/journal.pone.0133238

**Published:** 2015-07-29

**Authors:** Esther L. Calderon-Gierszal, Gail S. Prins

**Affiliations:** Departments of Urology and Physiology & Biophysics, College of Medicine, University of Illinois at Chicago, Chicago, Illinois, United States of America; University of Kentucky College of Medicine, UNITED STATES

## Abstract

Studies using rodent and adult human prostate stem-progenitor cell models suggest that developmental exposure to the endocrine disruptor Bisphenol-A (BPA) can predispose to prostate carcinogenesis with aging. Unknown at present is whether the embryonic human prostate is equally susceptible to BPA during its natural developmental window. To address this unmet need, we herein report the construction of a pioneer *in vitro* human prostate developmental model to study the effects of BPA. The directed differentiation of human embryonic stem cells (hESC) into prostatic organoids in a spatial system was accomplished with precise temporal control of growth factors and steroids. Activin-induced definitive endoderm was driven to prostate specification by combined exposure to WNT10B and FGF10. Matrigel culture for 20–30 days in medium containing R-Spondin-1, Noggin, EGF, retinoic acid and testosterone was sufficient for mature prostate organoid development. Immunofluorescence and gene expression analysis confirmed that organoids exhibited cytodifferentiation and functional properties of the human prostate. Exposure to 1 nM or 10 nM BPA throughout differentiation culture disturbed early morphogenesis in a dose-dependent manner with 1 nM BPA increasing and 10 nM BPA reducing the number of branched structures formed. While differentiation of branched structures to mature organoids seemed largely unaffected by BPA exposure, the stem-like cell population increased, appearing as focal stem cell nests that have not properly entered lineage commitment rather than the rare isolated stem cells found in normally differentiated structures. These findings provide the first direct evidence that low-dose BPA exposure targets hESC and perturbs morphogenesis as the embryonic cells differentiate towards human prostate organoids, suggesting that the developing human prostate may be susceptible to disruption by *in utero* BPA exposures.

## Introduction

The human prostate gland has a high incidence of abnormal growth and carcinogenesis with aging, contributing to extensive morbidity and mortality in men [1]. Despite considerable research, the basis for the high rates of prostate disease remains poorly understood. The prostate is derived embryologically from the endodermal urogenital sinus (UGS) which contrasts with the other male accessory sex glands that arise from the mesodermal Wolffian ducts [2]. Since seminal vesicle or vas deferens carcinoma is exceedingly rare, it has been postulated that the unique embryologic origin of the prostate gland contributes to its differential disease propensity [3]. Thus modeling prostate developmental events is an essential step towards understanding the basis of adult prostate disease.

It is well established that several hormones, including androgens and estrogens, play fundamental roles in normal prostate development and homeostasis and that imbalances in their level and activity contribute to aging-associated prostatic diseases [2]. Further, epidemiologic studies indicate that elevated estrogen levels *in utero* can predispose to an increased risk of prostate cancer later in life [4] which supports the paradigm of a developmental basis for adult disease. This is corroborated by considerable evidence using rodent models which determined that brief early-life exposures to exogenous estradiol can permanently reprogram the prostate gland, both structurally and epigenetically, and render it more susceptible to prostate cancer with aging [5–7].

In addition to natural estrogens, there is a rising concern regarding peri-natal exposures to endocrine disrupting chemicals (EDCs), many of which have estrogen-like actions. One prevalent endocrine disruptor, bisphenol A (BPA), initially synthesized in 1891, was identified as a synthetic estrogen in 1936 [8]. Today, BPA is a high-production chemical used in a wide range of consumer products including polycarbonate plastics, epoxy resins, carbonless paper receipts and dental sealants [9, 10]. Unfortunately, bioactive BPA monomers leach from these products, accumulate in the environment and are taken up by animals and humans [11–16]. Consequently, BPA and its metabolite BPA-glucuronide are detectable in the urine of most adults and children [16, 17], serum of pregnant women and newborns [18, 19], breast milk [20], amniotic fluid [21], cord blood [22, 23] and fetal livers [24]. Although BPA is rapidly metabolized to glucuronidated BPA (BPA-G) which lacks bioactivity and is excreted within 6–24 hour, free BPA has been detected in human sera at 0.2 to 1.0 ng/ml levels [22, 25]. While free BPA binds to nuclear estrogen receptors (ERs) with reduced affinity relative to 17β-estradiol [26], it possesses equivalent activational capacity for membrane ERs [27]. Thus, there is potential for this compound as a toxicant to developing, estrogen-sensitive human tissues.

Studies from our laboratory using a rat model found that transient neonatal exposure to low-dose BPA significantly increased the incidence of hormone-driven prostate carcinogenesis with aging [28] and modified the stem cell niche [29] which may underpin increased cancer susceptibility with aging. To test whether the human prostate may be similarly influenced, epithelial stem-progenitor cells were isolated from prostates of adult organ donors [30]. Exposure to estradiol or BPA activated membrane-initiated ER signaling, increased stem and progenitor cell proliferation, altered their transcriptome and modified noncoding RNA expression through histone modifications [31]. Further, when grafted into nude mice, transient exposure to low-dose BPA increased hormonal carcinogenesis in the human prostate epithelium [32]. Unclear at present is whether the human fetal prostate is equally susceptible to BPA exposures. Progress towards this goal has largely been impeded by restricted access to human fetal tissues and the lack of a suitable model for human embryonic prostate development.

During the past decade, several investigators have reported on prostatic models that utilize human embryonic stem cells (hESC) [33] and induced pluripotent stem cells (iPSC) derived from differentiated human prostate epithelium [34, 35]. Caveats of these models for examining fetal BPA exposures include the requirement of rodent mesenchyme for prostatic induction [33, 35] as well as the adult origin of the iPSC [34, 35]. Recently, advancements in stem cell biology have permitted directed differentiation of embryonic stem cells (ESCs) into a variety of organoids entirely *in vitro* thus providing robust models that provide unparalleled insight into the developmental processes of many organs [36, 37]. In this context, the goals of the present study were two-fold. First we sought to identify specific factors and conditions that would permit the *de novo* generation of three-dimensional (3-D) human prostate organoids *in vitro* through the directed differentiation of hESC. This was accomplished through temporal exposures to growth factors and steroids known to be essential for prostate development. Once established, this pioneer model was utilized to assess the effects of low-dose BPA exposures on the normal developmental process from the hESC stage to mature organoids. Results show that BPA exposure alters prostate organoid branching and expands the stem-cell like numbers in mature organoids, thus providing direct evidence that normal development of the human fetal prostate may be impaired by low-dose BPA *in utero*.

## Materials and Methods

### Directed differentiation of hESC to prostatic organoids

**Fig 1:**
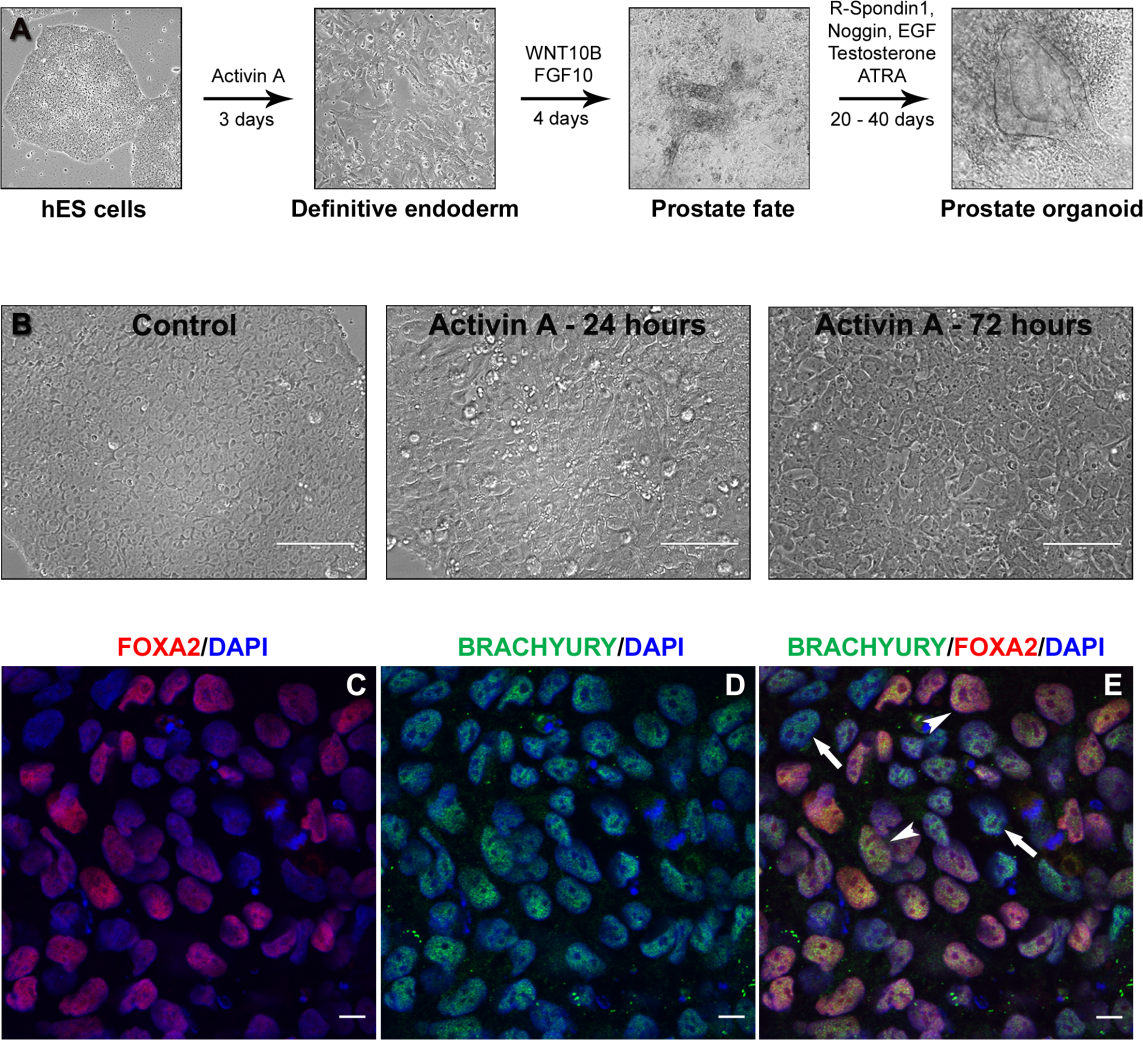
Model of human embryonic stem cell directed differentiation into prostate tissue *in vitro*. (**A**) Definitive endoderm and mesoderm differentiation was driven by culturing hESC with activin A (100 ng/ml) for 3 days. In the next 4 consecutive days, differentiated cells were cultured with WNT10B (500 ng/ml) and FGF10 (500 ng/ml) to direct them into prostatic fate and organoid formation. Organoids were transferred and grown in Matrigel to allow their 3-D growth in prostatic media containing T (1.7 μM) and ATRA (10 nM), which permits differentiation and expansion of prostatic organoids. (**B**) Definitive endoderm differentiation images show morphological changes at 24 and 72 hours following activin A treatment, compared to untreated hESC (control). Phase-contrast images were obtained using the EVOS microscope. Scale bars represent 200 μm. After 3 days of treatment with Activin A, Day 3 DE cultures were immunostained for (**C**) FOXA2 (red), an endodermal specific marker and (**D**) Brachyury (green), a mesendodermal marker with nuclear staining (DAPI, blue). The merged image (**E**) show definitive endoderm staining (red-yellow; arrowheads) in the majority of cells while a subpopulation was Brachyury^+^ but FOXA2^-^ (arrows) implicating a mesodermal component in a minority of cells. Scale bars represent 50 μm.

*In vivo*, the prostate gland originates from the endodermal urogenital sinus (UGS). Prostate determination occurs prior to morphologic evidence of a developing structure and involves sequential expression of molecular signals that commit a specific field of UGS cells to a prostatic fate [2]. Phenotypic prostate development commences as UGS epithelial cells form buds that penetrate into the surrounding mesenchyme that further instructs branching and differentiation. The presence of steroids, in particular testosterone and its metabolite DHT, is essential for prostate specification, development and functional maturation. In the present study, careful consideration was given to each of these features in an attempt to grow prostate organoids from hESC *in vitro*. Fig 1A provides a schematic summary that identifies the four key stages of prostate organoid culture in the current study; hESC colony culture, induction of definitive endoderm (DE), prostatic fate determination and prostate organoid growth and differentiation.

NIH-approved H1 (WA01), 46XY cells and H9 (H9inGFPhES), 46XX cells (WiCell Research Institute, Madison, WI) [38] were used with the approval of the University of Illinois at Chicago Embryonic Stem Cell Research Oversight Committee. Four-well Nunclon delta surface culture dishes (Nunc, Rochester, NY) and 6-well Falcon culture dishes (BD Biosciences, San Jose, CA) were used to thaw and expand cells, respectively. Both cell lines were grown on hES-qualified Matrigel (BD Biosciences) coated plates that permits growth in the absence of mouse embryonic fibroblast feeder cells. Cells were cultured in mTeSR1 medium (StemCell Technologies, Vancouver, Canada) at 37°C, with 5% CO_2_ and 95% humidity with fresh medium replenished every day. Cells were passaged every 4 days and differentiated regions were removed by scraping with pulled glass pipettes. To passage, hES cells were washed with DMEM/F12 (Gibco, Grand Island, NY), incubated with 1 mg/ml dispase (StemCell Technologies) for 5 minutes and gently washed three times with DMEM/F12. After the final wash, mTeSR1 was added and cells were lifted with a glass pipette and transferred to freshly Matrigel-coated plates.

To differentiate hES cells into DE, H9 or H1 cells were plated on plastic cover slips or 24-well Nunclon delta surface culture dishes (Nunc) and treated for 3 consecutive days with: 100 ng/ml activin A (R&D systems, Minneapolis, MN) in RPMI 1640 media (Gibco), 2 mM L-glutamine (Invitrogen, Grand Island, NY), 100 U/ml penicillin—100 μl/ml streptomycin (Invitrogen) and daily increasing concentrations of 0, 0.2 and 2% defined fetal bovine serum (Hyclone, Rockford, IL) as previously described [37].

Endoderm differentiated cells were driven into a prostatic fate by culture in 500 ng/ml human FGF10 (R&D systems) and 500 ng/ml human WNT10B (R&D systems) for 4 days in RPMI 1640 containing 2 mM L-glutamine and 100 U/ml penicillin—100 μl/ml streptomycin. Morphogen concentrations are similar to FGFs and WNTs previously used for directed differentiation of the intestine [37]. Freshly prepared prostatic fate media was changed daily. After 4 days, cells reached 100% confluence with visible 3-D structures attached to the bottom of the wells.

Organoids were carefully collected (~20–50/well), spun at 100 x g for 3 minutes and transferred in 25 μl to tubes with 50μl BD Matrigel Basement Membrane Matrix Growth Factor Reduced, Phenol Red-Free (BD Biosciences) containing 1X B27 supplement (Invitrogen), 100 ng/ml Noggin (R&D systems) and 100 ng/ml EGF (R&D systems). The matrigel/organoids mixture was pipetted onto 4-well Nunclon delta surface culture dishes and incubated for 20 minutes at 37°C to allow solidification. The resultant beads were covered with 500 μl of media containing 1:2 prostate epithelial cell growth medium (PrEGM) (Lonza) and stromal cell basal medium (SCBM) (Lonza) supplemented with 2 mM L-glutamine, penicillin-streptomycin, 15 mM HEPES (Gibco), 500 ng/ml R-Spondin1 (R&D systems), 100 ng/ml Noggin, 100 ng/ml EGF, 1X B27 supplement, 10 nM ATRA and 1.7 μM testosterone (Sigma-Aldrich Corp.). Freshly prepared media was changed every 4 days for ~30 days.

### In vitro BPA treatment

Crystallized BPA (National Toxicology Program, Research Triangle Park, NC) was dissolved in 100% EtOH. Cells were exposed to 1 nM, 10 nM BPA or EtOH vehicle (0.01% final concentration) throughout directed differentiation to prostate organoids. Each well received equivalent numbers of cells prior to endoderm differentiation to permit comparison of colony and organoid formation capabilities across treatment groups. Organoid cultures were imaged using a Zeiss Axiovert200 microscope with X-Y-Z stage and AxioCam for organoid quantitation and morphologic assessment. Numbers of budding, nonbudding and degenerative organoids as a function of BPA exposure were determined from images taken of all planes of representative areas for each treatment. Numbers were averaged/treatment/experiment and experiments replicated 6–9 times.

### Immunofluorescence and confocal imaging

Organoids were incubated with dispase (1 mg/ml) for 20 minutes at 37°C to partially dissolve Matrigel and 28 G needles were used to dissect individual organoids. Organoids or differentiated DE cells were fixed in 4% paraformaldehyde, washed with PBS, incubated with primary antibodies (Table 1) overnight at 4°C, rewashed with PBS and incubated with 1:200 dilution fluorochrome-conjugated secondary antibodies (Invitrogen) for 2 hours at room temperature. Following PBS wash, organoids were placed on raised chamber slides and mounted using Vectashield mounting medium with DAPI (Vector Laboratories Inc.). Confocal imaging was utilized to capture images from organoids and DE cells using a Zeiss LSM510 META microscope.

**Table 1:** Primary antibodies used for immunofluorescence.

Primary Antibody	Dilution factor	Company
Rabbit anti-AR (PG21)	1:100	[39]
Rabbit anti-Vimentin	1:100	Epitomics, Burlingame, CA
Rabbit anti-TMPRSS2	1:100	Epitomics, Burlingame, CA
Guinea pig anti-CK8/18	1:100	ARP American Research Products Inc., Waltham, MA
Goat anti-PSA	1:100	Santa Cruz Biotechnology Inc., Dallas, TX
Mouse anti-Laminin	1:100	LifeSpan BioSciences Inc., Seattle, WA
Mouse anti-NKX3.1	1:100	Novus Biologicals, Littleton, CO
Mouse anti-Trop2	1:200	Abcam, Cambridge, MA
Mouse anti-CD49f	1:200	Abcam, Cambridge, MA
Normal rabbit IgG	1:200	Kindly provided by Dr. Geofrey Greene, University of Chicago, Chicago, IL
Normal guinea pig IgG	1:300	Santa Cruz Biotechnology Inc., Dallas, TX
Normal goat IgG	1:100	Santa Cruz Biotechnology Inc., Dallas, TX
Normal mouse IgG	1:200	Zymed Laboratories Inc., San Francisco, CA

### RNA isolation and quantitative real-time PCR

RNA isolation was performed using TRIzol (Invitrogen) according to manufacturer’s instructions. One μg of total RNA was reverse transcribed into cDNA using iScript Reverse Transcription Supermix (Bio-Rad Laboratories Inc., Hercules, CA). One μl cDNA was combined with SsoAdvanced SYBR Green Supermix (Bio-Rad) to measure NKX3.1, AR, CK18, Vimentin, ERα, ERβ, GPER, TROP2, CD49f, p63, NANOG, OCT4 and RPL13 mRNA expression (Table 2). A CFX96 Real-Time System (Bio-Rad Laboratories Inc.) was utilized for quantification with the following conditions: preincubation at 95°C for 5 minutes, 40 cycles of denaturation at 95°C for 15 seconds, annealing at 60–63°C for 1 minute and extension at 95°C for 10 seconds. Data were analyzed by the comparative C_T_ method (–ΛΛC_T_) and mRNA levels were normalized to RPL13.

**Table 2:** Primer sequences used for real-time PCR.

Gene	Forward primer	Reverse primer
NKX3.1	5’-GGCCTGGGAGTCTTTGACTCCACTAC-3’	5’-ATGTGGAGCCCAAACCACAGAAAATG-3’
AR	5’-TGTCCATCTTGTCGTCTTCG-3’	5’-ATGGCTTCCAGGACATTCAG-3’
CK18	5’-CAGCAGATTGAGGAGAGCAC-3’	5’-TCGATCTCCAAGGACTGGAC-3’
Vimentin	5’-TTGACAATGCGTCTCTGGCAC-3’	5’-CCTGGATTTCCTCTTCGTGGAG-3'
ERα	5’-AAGCTTCGATGATGGGCTTA-3’	5’-AGGATCTCTAGCCAGGCACA-3’
ERβ	5’-AGTCCCTGGTGTGAAGCAAG-3’	5’-CATCCCTCTTTGAACCTGGA-3’
GPR30	5’-AATTCAAATGGCCAGTAGGG-3’	5’-TGGGTACCTGCCGTCCAGAT-3’
TROP2	5’- AGCTTGTAGGTACCCGGCGG-3’	5’- GTGTGCGCAAAAGGGAGGGG-3’
CD49f	5’-ACCAACACAGGTTCTCAAGG-3’	5’-ACCAACAGCAACATCAGG-3’
p63	5’-GTGAGCCACAGTACACGAACC-3’	5’-GAGCATCGAAGGTGGAGCTGG-3’
NANOG	5’-AATGGTGTGACGCAGAAGG-3’	5’-GGTTGCTCCAGGTTGAATTG-3’
OCT4	5’-AGGATGTGGTCCGAGTGTG-3’	5'-CAGAGTGGTGACGGAGACAG-3'
RPL13	5’-GTCTCCACGTGGTGTGTTTC-3’	5’-CAGGGCTTGGACTGTCTTTC-3’

### Statistical analysis

Data are expressed as mean ± SEM and analyzed using InStat version 3 (GraphPad software, Inc., San Diego, CA) using ANOVA followed by Student-Newman-Keuls or Bonferroni *post hoc* tests as appropriate to determine statistical significance.

## Results

### Directed differentiation of hESC into endoderm and mesoderm

hESC were first driven to DE by exposure to activin A, a Nodal mimic, for three consecutive days. Untreated cultures contained undifferentiated hESC characterized by compact growth of uniform multicellular colonies and a high nuclear-to-cytoplasmic ratio [38], whereas, activin A driven differentiation was apparent by 24 hours with loss of border integrity and uniformity (Fig 1B). Forty-eight hours after treatment, the monolayer of cells displayed a cobblestone appearance that persisted for 24 hours (Fig 1B). DE differentiation with 72 hours of activin A treatment was corroborated by double labeling immunofluorescence utilizing brachyury, a mesendodermal marker (precursor of mesoderm and DE) and the endodermal-specific marker, FOXA2 which co-localized in the majority of cells (Fig 1C–1E, arrowheads). In addition, a minor (~10–20%) subpopulation of cells was noted as brachyury^+^ but FOXA2^-^ (Fig 1E, arrows) indicating mesoderm differentiation which can generate the mesenchymal-derived stromal component of the prostate. Similar differential staining for a minor population of mesenchymal cells with a majority endodermal cells was previously shown in directed differentiation of hESC to intestine using this culture approach [37].

### Prostate fate determination

**Fig 2:**
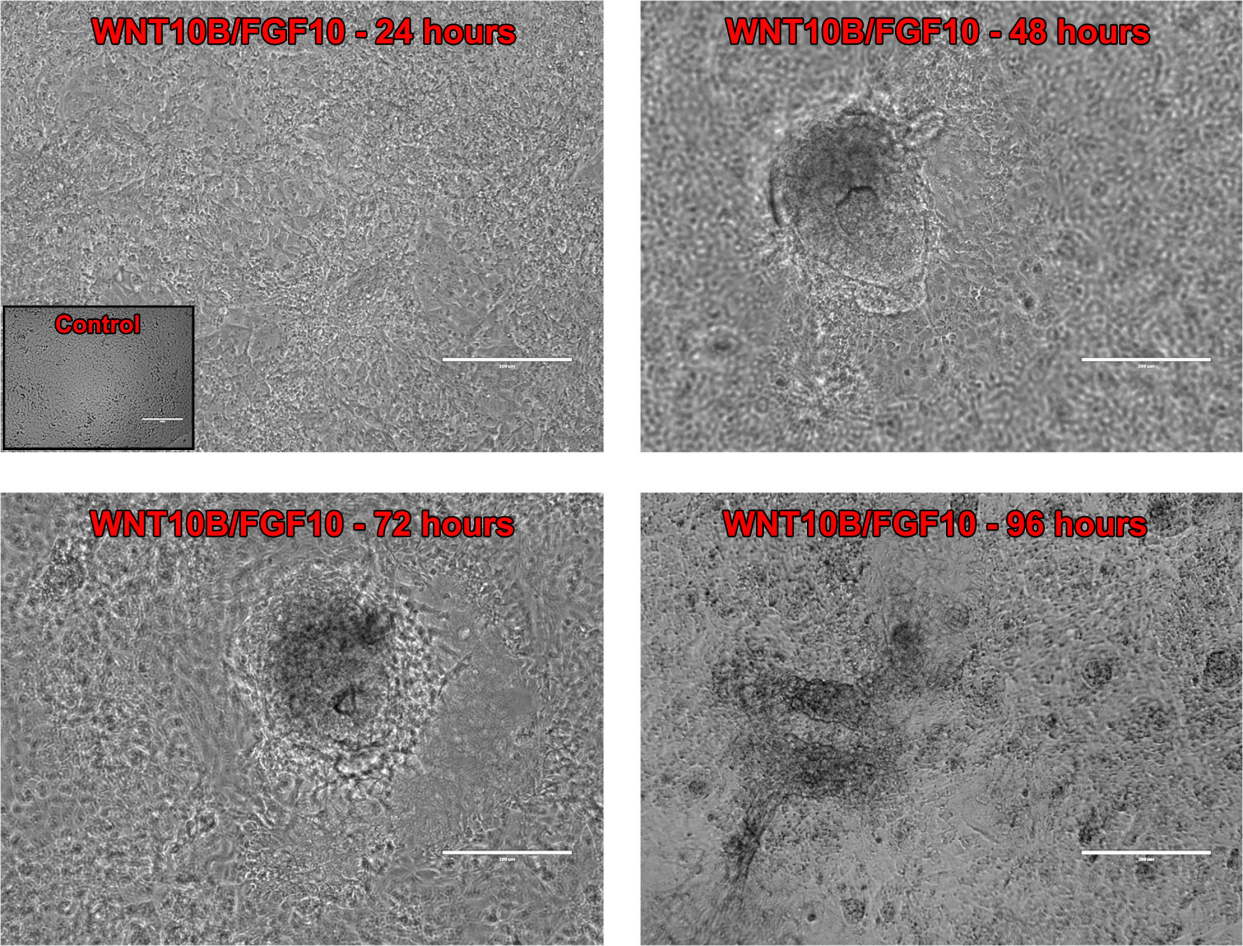
Directed differentiation into prostatic fate determination. Prostatic fate determination images illustrate morphologic changes over 96 hours of treatment with 500 ng/ml WNT10B and FGF10 compared to untreated hESC as control shown in inset. After 48 and 72 hours of growth factor culture, spheroid-like structures attached to the cell monolayer were observed. At 96 hours, prior to Matrigel culture, 3-D structures with a budding-like phenotype were observed. Phase contrast images were obtained using the EVOS microscope. Scale bars represent 200 μm.

Studies using ESCs to derive a variety of human tissues have shown that the coordinated temporal activation and repression of specific WNT and fibroblast growth factor (FGF) signaling pathways are essential for tissue specification and patterning. Hence, following endoderm differentiation, cells were driven into prostatic fate determination by culture in the presence of the secreted canonical WNT10B protein [40, 41], the earliest known secreted protein expressed by prostate epithelium immediately prior to bud formation [42], and FGF10, a urogenital mesenchyme-secreted growth factor essential for prostate epithelial budding and morphogenesis [43]. Four days of culture with both human growth factors was identified as the critical window for prostatic determination. Shorter culture was insufficient whereas exposure for 5–6 days in these morphogens markedly reduced organoid efficiency (data not shown). Notably, substitution of human WNT3A protein (500 ng/ml) was insufficient to drive prostatic structures (S1 Fig) which suggests a specificity for WNT10B actions that cannot be substituted by β-catenin activation through another canonical WNT. Similarly, culture in WNT10B or FGF10 protein alone was insufficient for derivation of differentiated prostate structures. At 24 hours of WNT10B and FGF10 treatment, the cell monolayer maintained a cobblestone-like morphology (Fig 2). Multilayers with attached 3-D spheroid-like structures were first observed at 48 hours and these continued to develop with 72 to 96 hours of culture with several spheroid-like structures giving rise to budding tubular-like structures (Fig 2).

### Prostate organoid development

**Fig 3:**
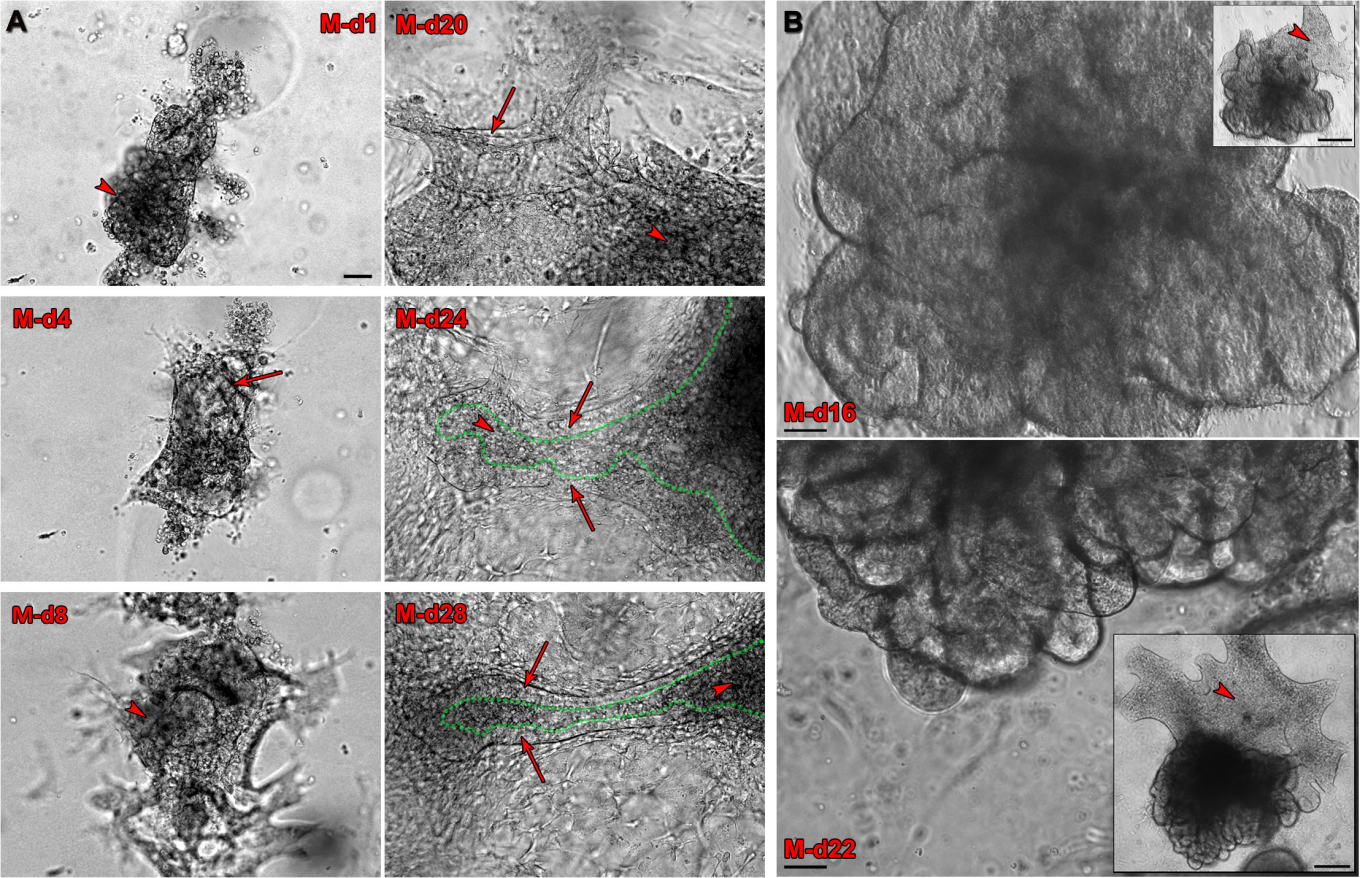
Characterization of prostatic phenotype. (**A**) Twenty-eight day time course phase-contrast images of a representative Matrigel cultured organoid forming over time from DE-differentiated H9 cells. While images of M-d1, d-4 and d-8 show the entire organoid as it grew, images of M-d20, d-24 and d-28 represent focal areas of formation and elongation of a single duct with extended culture. The representative duct is composed of a putative layer of columnar epithelium (arrows) with central lumens (dotted green lines), surrounded by mesenchyme (arrowheads). All images were obtained at the same magnification, scale bars represent 50 μm. (**B**) M-d16 and d-22 phase-contrast images following a representative Matrigel cultured organoid differentiated from H1 hESC. The organoid exhibited growth, budding, elongation and increased complexity over 6 days. Scale bars represent 50 μm. Insets: Lower magnification photographs show the entire organoid composed of convoluted ductal structures and mesenchyme (arrowheads). Scale bars represent 200 μm.

Following 4 days of specification culture, visible structures were collected and transferred to 3-D Matrigel culture for maturation to prostatic organoids. For this step, prostate specific media was developed and optimized to support branching morphogenesis and functional differentiation and included combined stromal and epithelial culture medias utilized for prostate cell culture, R-Spondin1 to potentiate endogenous Wnt signaling [44], Noggin to limit BMP signaling and permit branching morphogenesis [7], EGF to drive cell proliferation and augment budding, all-trans retinoic acid (ATRA) to enhance cytodifferentiation and testosterone (T), essential for prostate growth and differentiation [2]. In this system, the structures showed a rapid and steady increase in size, many exhibiting budding and outgrowth by Matrigel-day 8 (M-d8) (Fig 3A). An increase in the development of convoluted epithelial-like ducts with expanding morphologic complexity continued until harvesting on M-d28 (Fig 3A and 3B). Mature organoids at day 28–30 exhibited a complex network of epithelial-like ducts, composed of a layer of epithelium with a central lumen, surrounded by a basement membrane and stromal-type cells. No differences were observed between the H1 (XY) and H9 (XX) hESC lines in terms of capacity to produce differentiated organoids as assessed by morphology, immunostaining and RT-PCR for differentiation markers. While H9 is genetically XX, it is well established that the female UGS is fully capable of generating a functional prostate gland when exposed to T or DHT [45–47].

**Fig 4:**
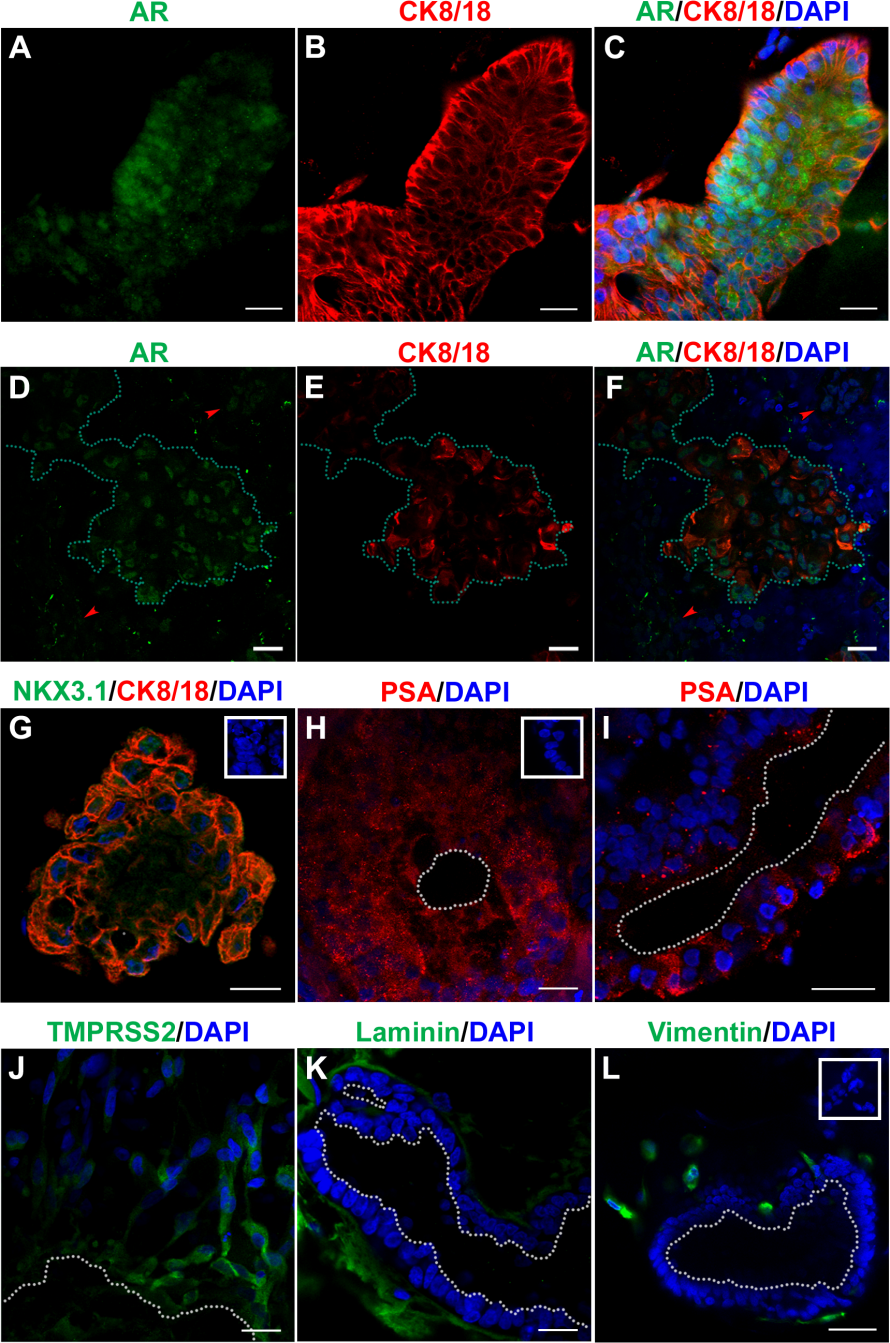
Characterization of functional differentiation of organoids. Immunostaining analysis by confocal microscopy of 28–30 day organoids for cytodifferentiation and functional differentiation markers. (**A and D**) Luminal cell cytodifferentiation markers AR (green) and (**B and E**) CK8/18 (red) and (**C and F**) merged images with DAPI (blue) reveals most luminal epithelial cells contain nuclear AR. (**G**) Merged NKX3.1 (green), a prostate specific epithelial cell marker, with CK8/18 (red), a luminal epithelial cell marker and DAPI (blue) shows nuclear NKX3.1 in all epithelial cells suggesting prostatic nature. Functional differentiation markers PSA (**H and I**) and androgen regulated gene TMPRSS2 (**J**) indicate the ability of cytodifferentiated luminal cells to produce secretory proteins specific to the prostate. Laminin (**K**), a basement membrane marker, delineates the normal acinar organization of the organoids. Staining for Vimentin (**L**) confirms the extra-acinar cells are derived from mesenchymally differentiated hESC cells, forming a stromal compartment. Normal IgG as negative controls for each probe is shown in insets. Lumens are indicated by white dotted lines (H-L) and epithelial ducts are outlined by green dotted lines (D-F). Scale bars represent 20 μm.

**Fig 5:**
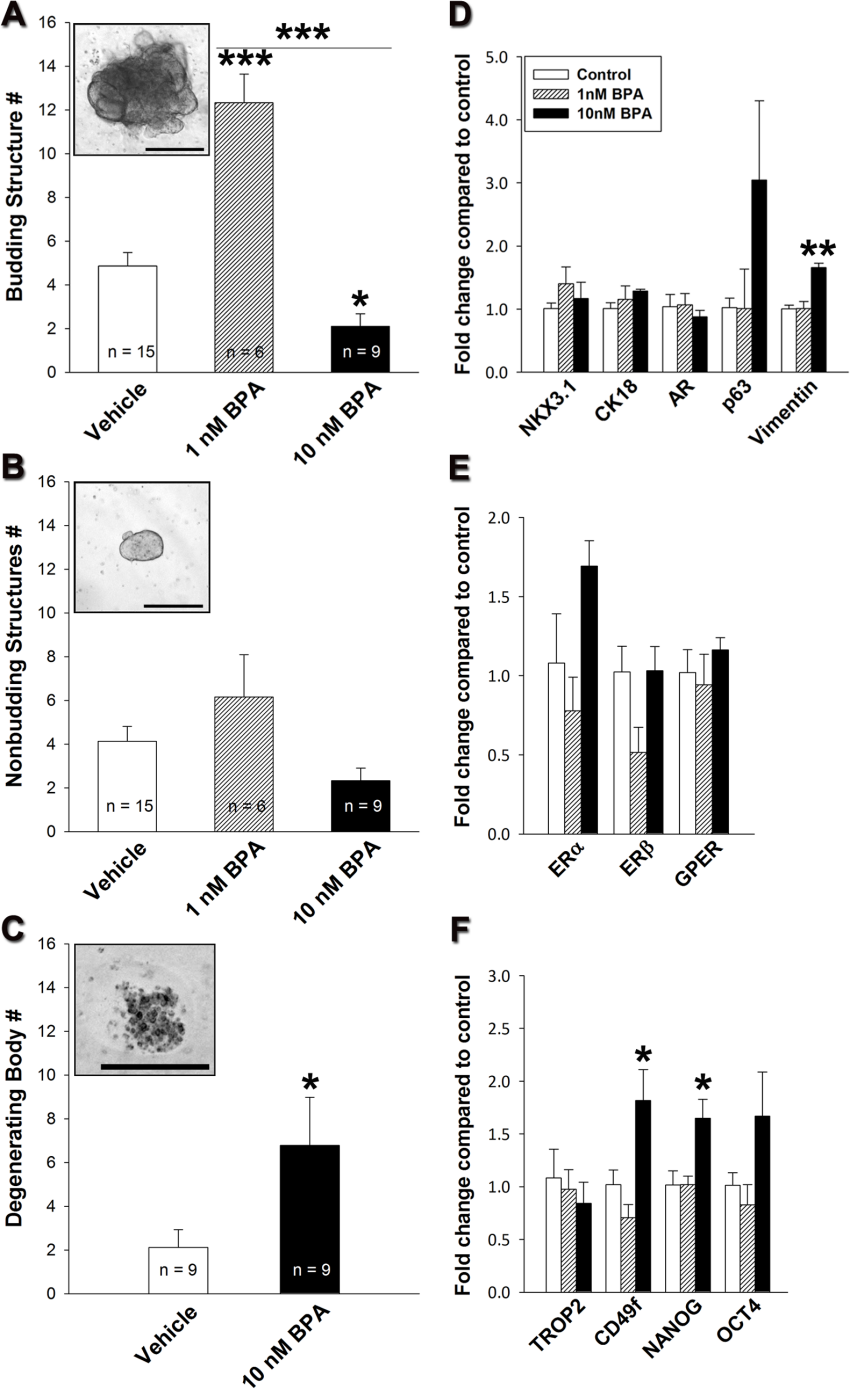
BPA effects during prostatic organoid development. Organoid quantitation (A-C) was performed 4 days following transfer to Matrigel in the absence or presence of 1 and 10 nM BPA. (**A**) 1 nM BPA increased budding (P < 0.001) whereas, 10 nM BPA reduced budding (P < 0.05) structure numbers compared to vehicle. (**B**) Nonbudding structure numbers were not affected by either dose of BPA. (**C**) While degenerating body numbers increased (P < 0.05) following 10 nM BPA exposure. (**D**) Differentiation gene expression of NKX3.1, CK18, AR and p63 was not altered by BPA treatment. In contrast, vimentin expression was significantly increased at 10 nM BPA (P < 0.01). (**E**) No difference in ERα, ERβ and GPER mRNA expression was noted. (**F**) Whereas, mRNA expression of 10 nM BPA treated organoids was significantly increased for the stem cell markers CD49f and NANOG (P < 0.05), with a similar trend noted for OCT4. TROP2 mRNA levels were not altered by BPA. From A-F bars represent means ± SEM (D-F n = 3), * P< 0.05, ** P < 0.01, *** P < 0.001. Scale bars represent 1 μm.

To evaluate and confirm that the differentiated organoids were prostatic in nature, mRNA for prostate epithelial genes and immunolocalization of several prostatic markers was performed using qRT-PCR and immunofluorescence confocal imaging on day 28–30 organoids. Protein marker examination (Fig 4A–4L) and gene expression analysis (Fig 5D–5F) documented epithelial and stromal cyto- and functional differentiation into prostatic-like structures resembling the adult human prostate gland. The luminal cells were marked by cytokeratin 8/18 (CK8/18; Fig 4B and 4E) and were largely androgen receptor (AR) positive (Fig 4A, 4C, 4D and 4F). Basal cell presence in all structures was indicated by p63 mRNA in all control cultures (Fig 5D). Their prostatic nature was confirmed by staining for multiple markers. NKX3.1 (Fig 4G), an epithelial transcription factor highly expressed in prostate epithelium and bulbourethral glands but not bladder, gut or seminal vesicles [48, 49], was found in all stained structures. TMPRSS2 (Fig 4J), an androgen-regulated proteases produced by human prostate epithelium was also found in all structures immunostained for this protein. Most importantly, prostate specific antigen (PSA; Fig 4H and 4I), another androgen-regulated protease produced exclusively by the prostate epithelium [50] was found in all immunostained structures, confirming the prostatic nature of the organoids. Laminin (Fig 4K), a basement membrane marker was used to delineate the normal acinar organization of the organoids which were surrounded by vimentin-positive stromal cells (Fig 4L). Some, but not all, of stromal-like cells were AR positive (Fig 4D, arrowheads) which is characteristic of prostate stroma [35]. Together, these data document the directed differentiation of hESC into functional prostatic organoids *in vitro* with operational capacity to respond to testosterone and produce secretory proteins.

### BPA effects on prostate organoid morphogenesis and differentiation

To directly assess the influence of chronic low-dose BPA exposure on human prostate morphogenesis and differentiation, the organoid cultures were exposed to vehicle, 1 or 10 nM BPA throughout the entire directed differentiation process. To evaluate effects on branching morphogenesis, budding and nonbudding structures were quantified 4 days following transfer to the 3-D Matrigel system. Exposure to 1 nM BPA significantly increased the number of budding structures while the number of nonbudding structures was not different from the control (Fig 5A and 5B). In contrast, treatment with 10 nM BPA significantly reduced the number of budding structures with limited effect on the nonbudding structures (Fig 5A and 5B). Further, while only occasionally noted in vehicle control and 1 nM BPA cultures, there was a marked increase (P<0.05) of degenerating structures in day 4 cultures exposed to 10 nM BPA (Fig 5C), appearing as membrane-bound structures containing apoptotic or degrading cells. No effect was noted on the size of the individual viable structures with either BPA concentration as compared to vehicle control cultures (data not shown). Due to the rapid development of branching complexity with continued organoid culture, comparisons of branching numbers and size were not possible at later time points.

**Fig 6:**
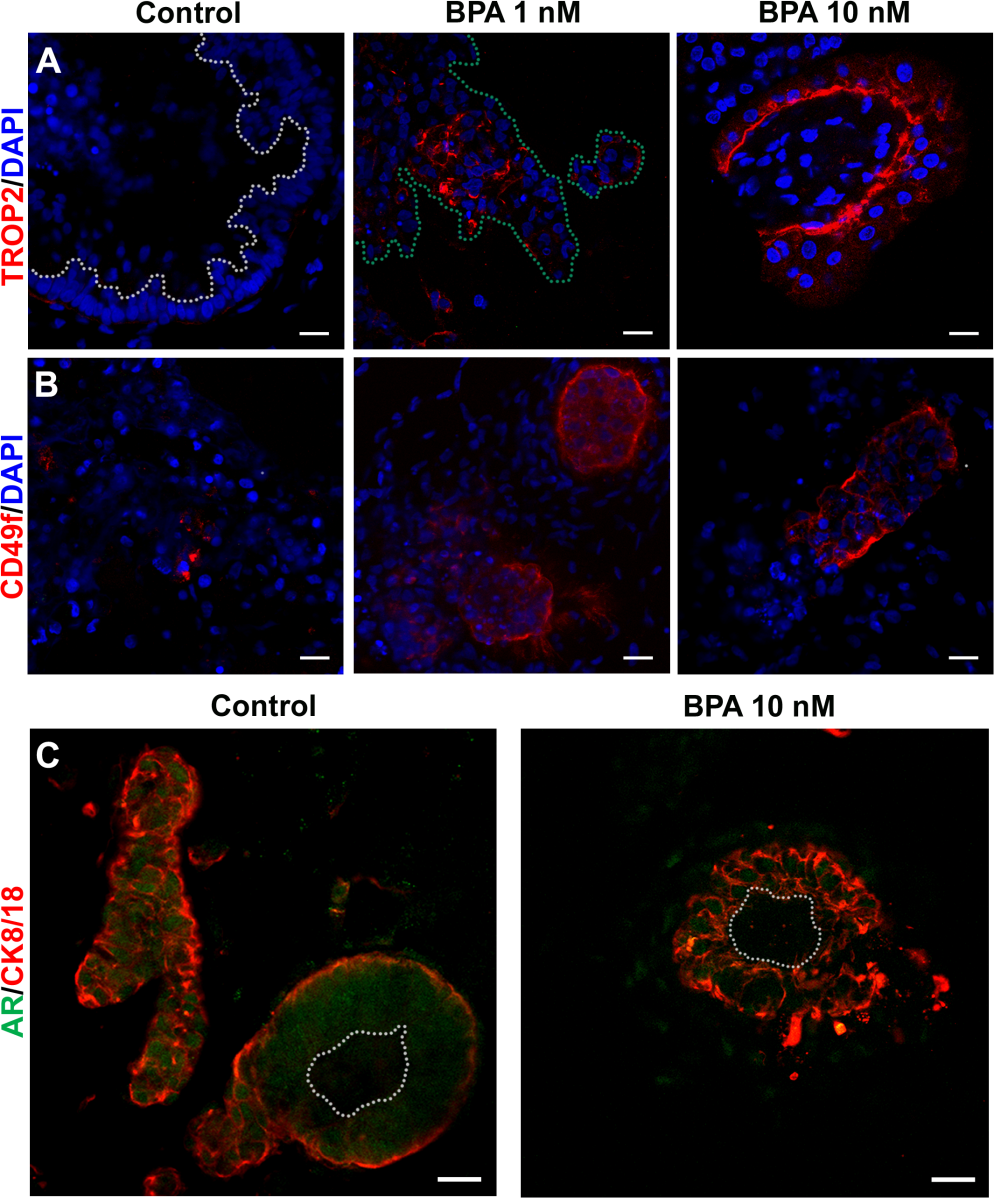
BPA effects during prostatic organoid maturation. Immunolocalization of the stem cell markers: (**A**) TROP2 and (**B**) CD49f show a dose-dependent increase in stem cell focal aggregates when treated with BPA compared to vehicle. (**C**) Organoids labeled with epithelial cytodifferentiation markers AR and CK8/18 show normal ductal morphology and epithelial differentiation after BPA treatment. Lumens are delineated by white and whole structures by green dotted lines. Scale bars represent 20 μm and all images are representative of n = 3.

To assess whether BPA affects cellular differentiation of the hESC into mature organoids, 3-D culture was continued to day 30 and cellular differentiation markers and steroid receptor expression were evaluated by qRT-PCR and immunofluorescence. Luminal epithelial cell differentiation markers NKX3.1 and CK18 as well as stromal and luminal AR levels were unaffected at the mRNA (Fig 5D) or protein levels (Fig 6C) by either 1 or 10 nM BPA as compared to vehicle controls. Interestingly, p63 mRNA was elevated by 10 nM BPA exposure, although this was not statistically significant (Fig 5D). While vimentin expression was not changed by 1 nM BPA, mRNA levels were significantly increased with 10 nM BPA exposure suggesting an increase in the stromal component by the higher BPA dose (Fig 5D). The expression of estrogen receptors (ERα, ERβ, GPER), confirmed mediators of BPA actions [30, 51], was not altered by developmental BPA exposure (Fig 5E).

In contrast to epithelial differentiation genes, the expression of the prostate epithelial cell stemness genes NANOG and CD49f within the whole organoids was significantly increased by 10 nM BPA (P < 0.05) as compared to vehicle or 1 nM BPA (Fig 5F) with a similar trend noted for OCT4. Gene expression for TROP2, another stem cell gene, remained unchanged by either BPA dose versus control organoids. Since stem cells comprise a very minor component of the mature prostate epithelium, the effect of BPA exposure on stem cells was examined by confocal immunolocalization using TROP2 and CD49f proteins as stem-like cell markers. For all control organoids, stem cell staining for TROP2 (Fig 6A) and CD49f (Fig 6B) revealed rare, sporadic single stem cells scattered within the epithelial compartment as is observed in the mature human prostate [52]. In striking contrast, exposure to either BPA dose resulted in a marked increase in numbers of TROP2^+^ and CD49f^+^ cells within the organoids (Fig 6A and 6B). Interestingly, these presented as focal stem cell aggregates or nests within the organoids rather than increased numbers of isolated stem cells throughout the structures. This is further illustrated by a Z-stack reconstruction video for TROP2 (S1 Video) in a 10 nM BPA exposed organoid that highlights a stem/progenitor niche that encircles one glandular structure. Together these images suggest that developmental BPA exposure may transiently increase symmetric self-renewal of stem cells in the developing organoids resulting in focal pockets of stem-like cell populations that fail to adequately commit to differentiated progeny.

## Discussion

This is the first report on the successful directed differentiation of hESC into human prostate organoids *in vitro*, recapitulating key prostate developmental events as they occur *in utero*. Activin A was utilized to promote DE differentiation [37], the lineage from which the prostate epithelium arises with a minor component of mesoderm differentiated cells. Together, this permitted the necessary cell lineages for subsequent differentiation towards prostate epithelium (endoderm) and stroma (mesoderm). It is known that the precise temporal addition of specific growth factors can give rise to different tissues e.g. liver, pancreas and hindgut from ESC [53]. In the present study, WNT10B and FGF10 were selected for directed differentiation of DE to prostate based on relevant scientific evidence from the rodent, the model from which prostate development has been deduced [2]. In the mouse, Wnt10b is expressed and secreted by specific UGS epithelial cells that form the prostate immediately prior to prostate bud initiation suggesting a specific role in prostate epithelial fate determination [54]. Fgf10, expressed and secreted by embryonic UGS mesenchymal cells, activates FGFR2iiic on adjacent epithelial cells and is essential for prostatic epithelial budding, ductal elongation and branching [43]. The present study identified the cocktail of WNT10B and FGF10 for a brief period to be sufficient to promote prostatic fate, as shown by the formation of spheroid-like structures prior to prostatic organoid formation in a 3-D milieu. Importantly, timing of WNT10B/FGF10 exposure, as occurs during fetal development, was crucial since shorter culture in these morphogens was insufficient to drive prostate determination whereas longer culture reduced organoid efficiency, thus precisely defining the window of specification. Furthermore, the substitution of the canonical WNT3A, which was sufficient to direct gut determination [34], impaired the formation of prostatic organoids, suggesting the essential and specific nature of canonical WNT10B for prostatic determination.

Growth and maturation of prostate organoids was accomplished by developing the appropriate prostate media to support both epithelial and stromal cell cyto- and functional differentiation. A combination of prostate epithelial cell media and stromal cell media was required since either medium alone was insufficient to support the growth of mature organoids. Several other exogenous factors necessary for budding, morphogenesis and organoid support were necessary for optimal organoid formation. R-Spondin1, a Wnt agonist, was used to enhance endogenous Wnts known to be expressed in a spacio-temporal fashion by the prostate during development and maturation [44]. Noggin was utilized to antagonize the negative regulation of BMP on prostate growth, a process necessary for branching morphogenesis [7]. EGF was added to regulate budding and promote proliferation of mesenchymal and epithelial cells [55]. ATRA was utilized to drive cytodifferentiation and testosterone was added as the essential androgen required for prostate development and functional differentiation. Testosterone rather than DHT was used to allow its aromatization to estradiol which is necessary for normal prostate development [56, 57]. Under these conditions prostatic organoids grew in size, underwent branching morphogenesis and formed a complex network of epithelial-like ducts, composed of a single layer of columnar epithelium with central lumens, surrounded by a basement membrane and stromal cells as observed in the human prostate. Marker analysis revealed the prostatic nature of the organoids obtained by expression of NKX3.1, AR, TMPRSS2 and PSA. Together, the present data support the construction of a human model for prostate development *in vitro* that contains entirely human stromal and epithelial components and possesses similar architecture and functional properties found in the human prostate.

Utilizing this novel model, the current study provides direct evidence that low levels of BPA (1 and 10 nM) can target the hESC, disturb human prostate morphogenesis and perturb prostate stem cell homeostasis in maturing prostate structures. Interestingly, exposure to 1 nM BPA during early morphogenesis augmented whereas 10 nM BPA decreased the number of budding and total structures formed in 3-D Matrigel culture at day 4. The bud-promoting effects at the 1 nM dose support previous studies using murine embryonic urogenital sinus culture which found an increase in prostatic duct formation and androgen receptor binding with exposure to 0.2 nM BPA as compared to vehicle [58]. While a wider range of exposures will be necessary to define a complete dose-response analysis, the preliminary biphasic response to 1 and 10 nM BPA supports multiple previous reports on non-monotonic dose-responses for BPA in rodent prostates, human prostate cancer cells and other organ systems with low doses stimulating and higher doses inhibiting organ size and cell proliferation [59, 60]. Evidence for mild toxicity from 10 nM BPA was apparent by a significant increase in the number of degenerating bodies at day 4 which suggests that early developing structures may be particularly sensitive to continuous BPA exposure, even at this relatively low dose. It is important to note that human fetal exposures to BPA, determined by monitoring mid gestational umbilical cord blood, found a geometric mean of 0.16 ng/ml free BPA (0.64 nM) with a small subset of fetuses having high levels of unconjugated BPA (>18 ng/ml) [22]. Thus the two BPA doses used in the present study are considered environmentally relevant to human fetal exposures. It is possible that variable prostatic BPA effects may ensue in humans depending on the BPA exposure level experienced *in utero* with lower doses augmenting prostate growth and branching and higher exposures driving apoptotic responses and limiting prostate growth.

Continued exposure of the viable structures to BPA as they developed to mature prostatic organoids resulted in disruption of stem cell homeostasis as evidenced by increased mRNA expression of CD49f, NANOG and OCT4 within whole organoids exposed to 10 nM BPA. Analysis of protein localization for TROP2 and CD49f by immunofluorescence provided phenotypic insight by revealing an increase of focal pockets of stem cell aggregates positive for TROP2 and CD49f within the organoids exposed to either BPA dose with the greatest effects seen at 10 nM BPA. This is in marked contrast to the control organoids and normal mature prostate epithelium where rare stem-like cells appear as single cells intermittently localized near the basement membrane. It is believed that epithelial homeostasis is derived and maintained in mature tissues, including the prostate, through symmetric and asymmetric cell division of rare stem cells that, respectively, maintain their self-renewal capability and produce progenitor cells which differentiate to epithelial cell lineages [61]. These events are tightly regulated by the stem cell niche through secreted growth factors, hormones and cell-cell communication [62]. Herein a mechanism is proposed whereby BPA exposure can transiently augment prostate stem cell symmetric division and repress asymmetric division and/or proper entry into lineage commitment in the maturing prostatic organoid resulting in the accumulation of focal stem cell nests throughout the structures. This is supported by our recent study which demonstrated that BPA exposure increased adult prostate stem-progenitor cell proliferation *in vitro* [32] and restrained entry into differentiation pathways in a timely manner. Furthermore, recent findings in murine adipocytes [63], neural cells [64], mammary gland [65] spermatogonia [66] and human umbilical cord blood showed an increase in the stem cell pool following exposure to BPA [67] and/or other EDCs including diethylhexylphthalate, tributyltin, and BPA diglycidyl ether [63]. Since stem cell numbers in diverse organs have been shown to influence carcinogenic susceptibility [68], we hypothesize that stem cell perturbations, as observed herein as a function of developmental BPA exposures, may underpin an increased risk of human prostate cancer with aging, as has been observed in animal models.

## Conclusions

The present work reports the construction of a pioneer human model for prostate development *in vitro* by the directed differentiation of hESC into prostatic organoids and demonstrates a previously unidentified key role of WNT10B in prostate epithelial specification. This model possesses analogous architecture and functional properties found in the human prostate that can be applied in future biomedical research. Importantly, this *in vitro* model may provide considerable utility to evaluate the effects of other EDCs, screen for chemical toxicity and assess potential adverse or beneficial effects of pharmaceutical compounds on the developing and mature prostatic structure.

The relevance of our model was shown by its efficacy in addressing the critical question of whether the human fetal prostate is affected by BPA exposures which has not been previously determined due to the lack of appropriate models. The present findings document for the first time that low-dose BPA can target the hESC, disrupt human embryonic prostate morphogenesis and perturb prostate stem cell homeostasis in a maturing prostate structure.

## Supporting Information

S1 Fig Phase contrast images of Matrigel d-28 organoids exposed to WNT3A-FGF10. Definitive endoderm cells were cultured for 4 days in the presence of WNT3A (500 ng/ml) plus FGF10 and transferred to Matrigel culture for 28 days in growth medium as detailed in Materials and Methods. In contrast to complex branched structures observed after directed differentiation with WNT10B plus FGF10, the resultant organoids that remained after WNT3A-FGF10 exposure were small spheroids that failed to grow and branch. This suggests an essential requirement for WNT10B which is not replicated by canonical WNT activation alone. Scale bars represent 50 μm.
(TIF)

S1 Video TROP2 Z-stack reconstruction video. QuickTime movie of Z-stack from immunofluorescently labeled prostate organoid with stem cell marker TROP2 (red) and DAPI (blue) illustrating a focal stem cell cluster. Z-stack contains 51 planes at 1.8 μm intervals along the Z axis.
(MOV)
