# Supplementary figures and images for: Directed Differentiation of Human Embryonic Stem Cells into Prostate Organoids In Vitro and its Perturbation by Low-Dose Bisphenol A Exposure
Source: PLoS One. 2015 Jul 29;10(7):e0133238. doi: 10.1371/journal.pone.0133238 (PMC4519179; doi:10.1371/journal.pone.0133238)

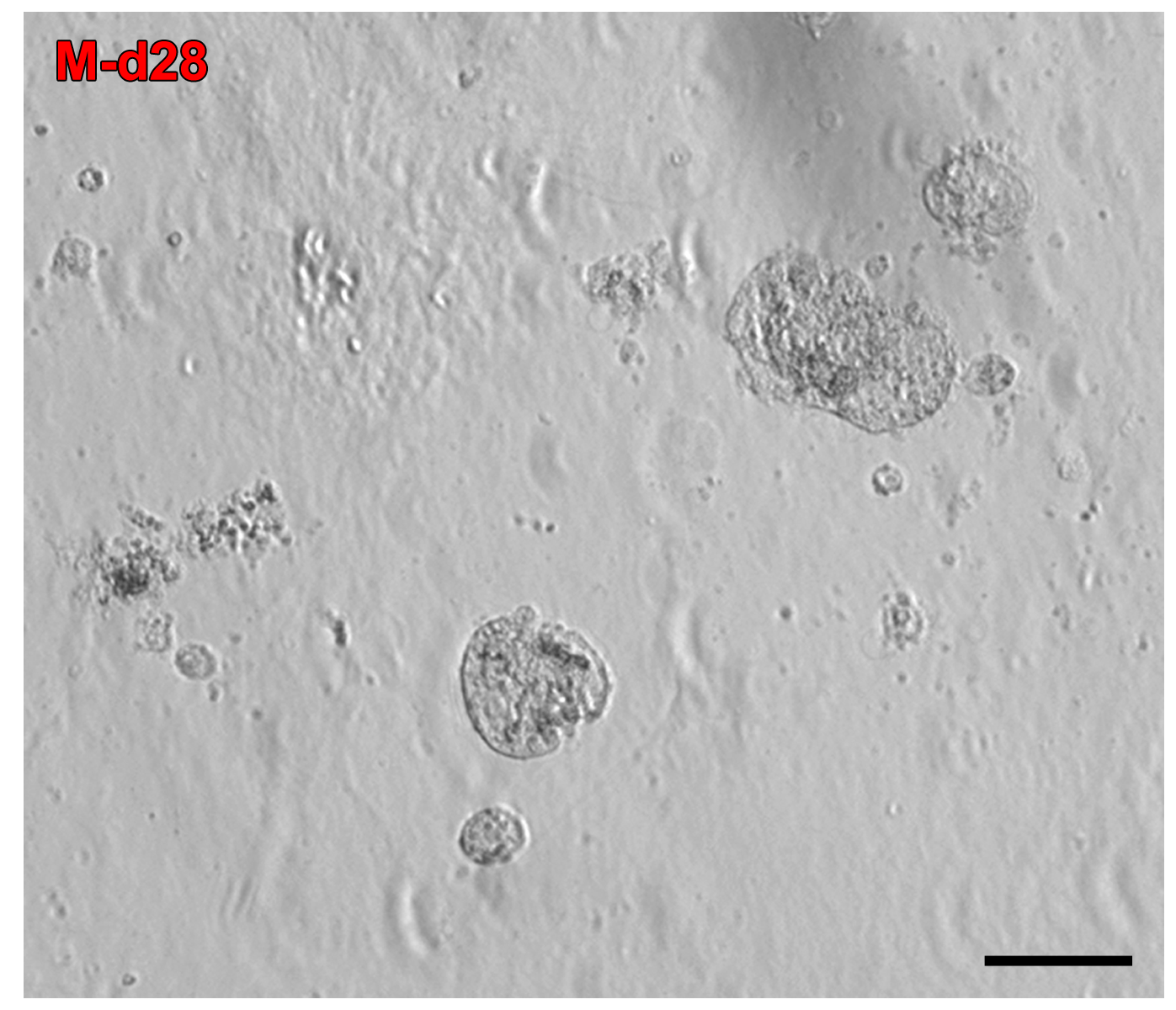

Supplement: S1 Fig — Definitive endoderm cells were cultured for 4 days in the presence of WNT3A (500 ng/ml) plus FGF10 and transferred to Matrigel culture for 28 days in growth medium as detailed in Materials and Methods. In contrast to complex branched structures observed after directed differentiation with WNT10B plus FGF10, the resultant organoids that remained after WNT3A-FGF10 exposure were small spheroids that failed to grow and branch. This suggests an essential requirement for WNT10B which is not replicated by canonical WNT activation alone. Scale bars represent 50 μm. (TIF) [file pone.0133238.s001.tif]
